# Cognitive Multitasking: Inhibition in Task Switching Depends on Stimulus Complexity

**DOI:** 10.5334/joc.115

**Published:** 2020-09-29

**Authors:** Stefanie Schuch, Otmar Bock, Klara Freitag, Luca Moretti

**Affiliations:** 1 Institute of Psychology, RWTH Aachen University, Aachen, DE; 2 Institute of Exercise Training and Sport Informatics, German Sport University, Cologne, DE

**Keywords:** task inhibition, N-2 repetition costs, stimulus complexity, ecological validity

## Abstract

We report a series of three experiments investigating inhibition in task switching, using N-2 repetition costs as an empirical marker. The experiments were structurally identical, employing a standard experimental paradigm where participants switch between three different categorization tasks. The experiments differed with respect to the stimulus material. According to prominent theories of cognitive control, N-2 repetition costs should be observed in all three experiments. To our surprise, this is not what we observed: N-2 repetition costs did not occur in Experiment 1, where we used static pictures from a driving simulator environment showing an oncoming car, embedded in a car-driving scene. In contrast, we observed robust N-2 repetition costs in Experiment 2, where we used static pictures of faces, and in Experiment 3, where the identical car stimuli from Experiment 1 were used, but without the surrounding visual scene. These results suggest that N-2 repetition costs depend on the complexity of the stimulus material. We discuss two aspects of complexity: 1) When the relevant stimulus feature is embedded in a complex visual scene, task-irrelevant features in that scene might trigger additional task sets, and thus induce additional task switches, attenuating N-2 repetition costs among the instructed task sets. 2) The presence of distractors might lead to additional covert or overt shifts of spatial attention, which in turn might reduce the size of N-2 repetition costs. On a more general level, the results illustrate the difficulty of transferring laboratory tasks to settings that bear more similarity to everyday life situations.

## Introduction

In cognitive psychology, paradigms have been developed to investigate cognitive processes in constrained, minimalized laboratory settings, in order to isolate the cognitive processes in question. It is often assumed that findings from laboratory studies can serve to explain human behavior in everyday life situations outside the lab. However, this assumption is not always valid: Laboratory and daily life settings differ in a variety of aspects (e.g., Bock & Hagemann, 2010; Bock et al., 2019), and empirical effects observed in a laboratory context cannot always be transferred to contexts outside the lab. For example, substantial differences between performance on classical laboratory tasks on the one hand, and performance on everyday or everyday-like tasks on the other hand, have been observed for locomotion (Bock & Beurskens, 2010), for manual grasping (Bock & Züll, 2013; Steinberg & Bock, 2013) and its age-related decay (Bock & Baak, 2013; Bock & Steinberg, 2012), as well as for age-related changes of cognitive skills (Verhaeghen, Martin, & Sêdek, 2012). In general, the problem of ecological validity of laboratory findings is repeatedly discussed in applied research contexts, such as neuropsychological and clinical contexts (Dawson & Marcotte, 2017), memory research (Kvavilashvili & Ellis, 2004), child development (Schmuckler, 2001), and ageing research (Bock & Steinberg, 2012; Verhaeghen et al., 2012; Wollesen & Voelcker-Rehage, 2014).

Here, we report a series of experiments showing that a robust empirical effect from laboratory research on multitasking is not robust anymore when the stimulus material becomes more complex, and hence more naturalistic, even when still inside the lab. We systematically manipulated aspects of stimulus material and stimulus presentation, in order to determine possible causes for these discrepancies.

We focus on an empirical marker of inhibitory control in multitasking situations, which is called “N-2 task repetition costs” (Mayr & Keele, 2000; see Koch, Gade, Schuch & Philipp, 2010, for review). These costs can be measured in a task-switching paradigm where participants rapidly switch between different cognitive tasks. N-2 repetition costs denote the finding that reaction times in task A are longer when A is the third member of a sequence …AB*A…* (N-2 task repetition sequence), rather than the third member of a sequence …CB*A…* (N-2 task switch sequence). The reasoning is that during the switch from task A to task B, task A becomes inhibited, and this inhibition decays slowly over time. Therefore, the sooner one switches back to the previously inhibited task A, the more persisting inhibition needs to be overcome, leading to worse performance in AB*A* (switching back to task A after just one intermediate trial) than CB*A* sequences (switching back to task A after at least two intermediate trials; Koch et al., 2010; Mayr & Keele, 2000; Sexton & Cooper, 2017).

All of the experiments reported here are structurally very similar. In all experiments, participants switch between three different categorization tasks. In every trial, a static visual stimulus is presented that has to be categorized according to one of three tasks. In Experiments 1 and 3, participants categorize the picture of a car according to 1) the color, 2) the brand, or 3) the presence of a toll sticker on the windscreen. In Experiment 2, participants categorize pictures of human faces according to 1) the age, 2) the gender, or 3) the emotional expression of the presented face. In both paradigms, the relevant task cannot be inferred from the presented stimulus, because every car can be categorized according to color, brand, or toll sticker, and every face can be categorized according to age, gender, or emotional expression (i.e., the stimuli are “multivalent”; see Kiesel et al., 2010; Monsell, 2003; Vandierendonck, Liefooghe, & Verbruggen, 2010; for overviews of task-switching methodology). Therefore, the relevant task (i.e., the relevant categorization rule) is indicated by a task cue in all experiments. Also, in both paradigms, the same set of response keys is used for the different tasks, leading to overlapping category-response rules in all experiments (i.e., the responses are multivalent; see Monsell, 2003; Kiesel et al., 2010).

## Experiment 1

In Experiment 1, we assessed N-2 repetition costs in a paradigm resembling a car-driving situation, where the oncoming car needs to be categorized according to color, brand, or the presence of a toll sticker. We also varied the type of task cue, with one group of participants receiving auditory verbal cues (the spoken words “color”, “brand”, “toll sticker”, in German); a second group receiving visual verbal cues (the printed words “color”, “brand”, “toll sticker”, in German); and a third group receiving visual abstract cues (frames around the pictures, with different boldness of the frame lines). The type of task cue has been shown to influence the size of task-switch costs, and of N-2 repetition costs, in task switching (see Jost, De Baene, Koch & Brass, 2013, for review).

We reasoned that N-2 repetition costs should be larger with visual than with auditory cues, and larger with abstract than with verbal cues. Cue modality (i.e., auditory versus visual) has been reported to influence task-switch costs (e.g., Kreutzfeldt, Stephan, Willmes & Koch, 2017; Lukas, Philipp, & Koch, 2010), and might influence N-2 repetition costs as well. In particular, visual cues might interfere more than auditory cues with the visual scenes of car driving presented here (Graham, 1999; Ho & Spence 2005), possibly leading to more interference between the task sets, and hence larger N-2 repetition costs. Within the two visual cue conditions, the abstract shape cues might induce larger N-2 repetition costs than the visual word cues, because with the abstract cues, participants need to establish new associations between cue and task-set (e.g., “frame with thin lines indicates color task”), whereas with the visual word cues, they can draw upon pre-existing semantic associations when processing the cues (Arbuthnott & Woodward, 2002; Logan & Schneider, 2006; Mayr & Kliegl, 2000). N-2 repetition costs have been shown to be larger with abstract cues than with cues that have pre-existing associations with the task-set (Grange & Houghton, 2010; Houghton, Pritchard, & Grange, 2009; see also Gade & Koch, 2007, 2008).

### Method

#### Participants

36 persons participated, 12 in each cue condition. 31 were female, 5 male; mean age was 21.8 years; all were between 18 and 37 years of age. They were either students, or friends of students, of RWTH Aachen University. They received either monetary compensation (6 Euros), or course credits, or sweets, in return for participation.

#### Stimulus material

**Figure 1:**
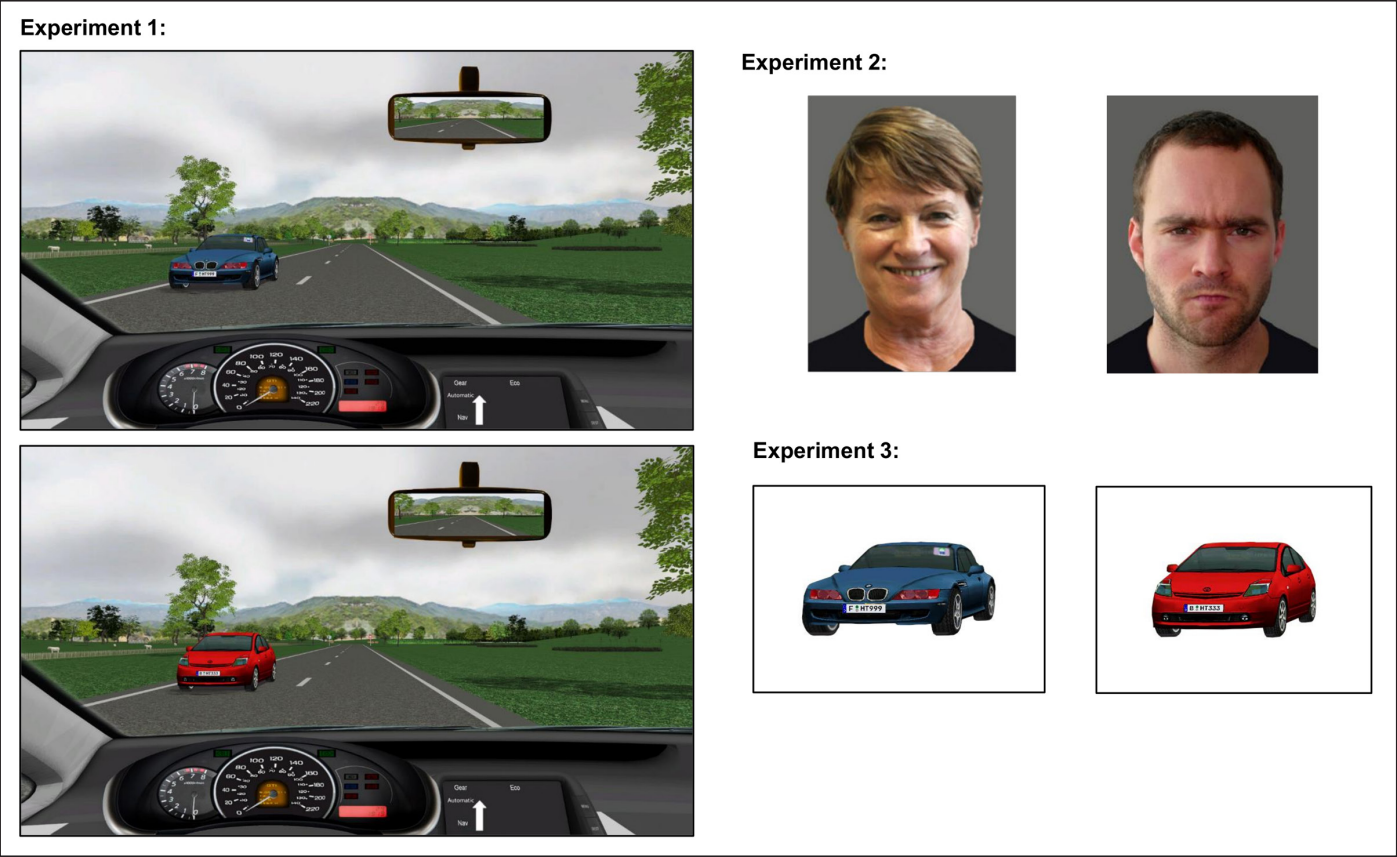
Example stimuli used in Experiments 1, 2, and 3. Left side: Experiment 1. Stimulus size was 29.5 cm by 19.3 cm (28.5 by 16.0 cm in Experiment 1c). The stimuli differed only with respect to features of the oncoming car. In the upper example, the oncoming car is a blue BMW with a toll sticker on the windshield; in the lower example, the oncoming car is a red Toyota Prius without toll sticker. Right side, upper: Experiment 2. Stimulus size was 10.5 cm by 14.0 cm. An old female happy face and a young male angry face are presented as examples. Right side, lower: Experiment 3. Stimulus size was 14.0 cm by 10.5 cm. The oncoming cars from the pictures of Experiment 1 were presented centrally on plain background.

The stimuli were eight static pictures (i.e., screenshots) taken from a driving simulator environment (Carnetsoft© BV, Groningen, NL). All pictures presented a view through the windshield of a car, with an oncoming car in the left lane (see Figure 1 for examples). The pictures differed with respect to the oncoming car, which could be colored red or blue, be of brand “BMW” or “Toyota Prius”, and have a toll sticker present or absent on the windscreen. In total, eight different pictures were used, one for each possible combination of color (red/blue), brand (bmw/prius), and toll sticker (present/absent). Additionally, the license plate of the oncoming car varied across the eight different pictures, but this was not relevant for any of the tasks. The pictures were of width 29.5 cm and height 19.3 cm in Cue Conditions 1a and 1b, and of width 28.5 cm and height 16.0 cm in Cue Condition 1c (because here, the task cues were frames around the pictures, see below). Viewing distance was about 40 cm.

#### Task cues

Three different kinds of task cues were employed in Cue Conditions 1a, 1b, and 1c. In Cue Condition 1a (N = 12), participants received verbal cues over headphones (Sennheiser, model PMX 95). The spoken words “Farbe”, “Marke”, “Vignette” (which are the German words for color, brand, and toll sticker, respectively) were presented. The total length of each sound file was 2000 ms; the word “Farbe” was fully produced after 980 ms, “Marke” after 1030 ms, “Vignette” after 1030 ms.

In Cue Condition 1b (N = 12), participants received visual verbal cues. The words “Farbe”, “Marke”, or “Vignette” were presented centrally on the screen in white font on black background, with font size 40.

In Cue Condition 1c (N = 12), participants received visual abstract cues. These were centrally presented white frames, with the inside of the frame measuring 28.5 cm in width and 16.0 cm in height (i.e., the size of the car pictures, which were slightly smaller in 1c than in 1a and 1b). The frames differed with respect to the thickness of the horizontal and vertical lines. The color task was indicated by a frame with horizontal and vertical lines of thickness 0.3 cm each; the brand task by a frame with horizontal lines of thickness 1.0 cm and vertical lines of thickness 0.3 cm; the toll-sticker task by a frame with horizontal lines of thickness 0.3 cm and vertical lines of thickness 1.0 cm.

#### Responses and response mappings

Participants responded by pressing the left or right response key (the “x” and “,” keys on a German QWERTZ computer keyboard) with their index fingers. The category-to-response mappings were counterbalanced across participants in each cue condition. For half of them, the left key meant “red”, “bmw” or “sticker” and the right key meant “blue”, “prius” or “no sticker”, for the other half the sides were reversed. A paper card displaying the response mappings was placed above (Cue Condition 1a) or below (1b, 1c) the screen and remained visible throughout the experiment.

#### Procedure

All participants were tested individually in a quiet room. Instructions were presented on the screen, and any questions were answered by the experimenter. Participants were encouraged to respond as quickly and accurately as possible. The experimenter stayed in the room during the practice session (1 block of 30 trials), and left the room for the experimental session (24 blocks of 30 trials each, separated by short self-paced breaks). The experiment took about 45 minutes in total.

The relevant task varied from trial to trial in a pseudo-random sequence with the following constraints: Task repetitions from trial N-1 to trial N were not allowed, neither were stimulus repetitions from trial N-2 to trial N, or from trial N-1 to trial N. Across all blocks, N-2 task repetitions and N-2 task switches occurred equally often; each of the three tasks occurred equally often with each stimulus, and with a left or right response.

Each trial started with a cue indicating the relevant task, followed by a car stimulus that had to be categorized by pressing the left or right key. In case of a wrong response, an error feedback occurred. The exact trial procedures differed slightly between Cue Condition 1a (auditory verbal cue) and Cue Condition 1b & 1c (visual verbal cue and visual abstract cue, respectively).

In Cue Condition 1a, the time interval between onset of sound file (cue) and onset of car picture (stimulus) was 2000 ms (cue-stimulus interval, CSI). After stimulus onset, participants had 2000 ms to respond. If the correct response was given during this interval, the picture disappeared immediately, and the next task cue was presented after 400 ms. If an incorrect response was given during this interval, the picture disappeared immediately, and an auditory verbal error feedback (“Fehler”) was presented for 400 ms. If no response was given within 2000 ms of stimulus onset, participants received an auditory verbal feedback signaling a too slow response (“zu spät”), and the next task cue was presented 2000 ms after onset of the too-slow feedback.

In Cue Conditions 1b and 1c, the CSI was 500 ms. The task cues were visually presented words or frames, respectively, and disappeared at the onset of the car picture. There was no time limit for responding, in order to discourage fast guesses. As soon as the correct response key was pressed, the picture disappeared, and after 1000 ms, the next task cue was presented. When the wrong response key was pressed, an error feedback occurred for 1000 ms, and after another 1000 ms, the next task cue was presented.

#### Design

A 2 × 3 mixed design was applied with the within-subject variable Task Sequence (N-2 Repetition, N-2 Switch) and the between-subject variable Cue Condition (1a: auditory verbal cues, 1b: visual verbal cues, 1c: visual abstract cues). We also conducted post-hoc *t*-tests to establish whether N-2 repetition costs could be observed within each cue condition; all *t*-tests were two-tailed. The dependent variables were mean reaction times (RT) and error rates (ER) in all experiments.

### Results

#### Raw data

The raw data of all experiments are available on the Open Science Framework: https://osf.io/jez7h/.

#### Data filtering

The first two trials per block, the two trials following an error, and outliers (defined as trials with RT deviating at least 3 SDs from an individual’s mean RT; 1.7% of all trials), were excluded. For RT analysis, error trials were excluded as well. In Experiment 1, the mean number of trials per condition and participant included in RT analysis was 276 (minimum 163, maximum 324); the mean number included in analysis of Error Rates was 292 (min 202, max 326).

#### RT

**Figure 2:**
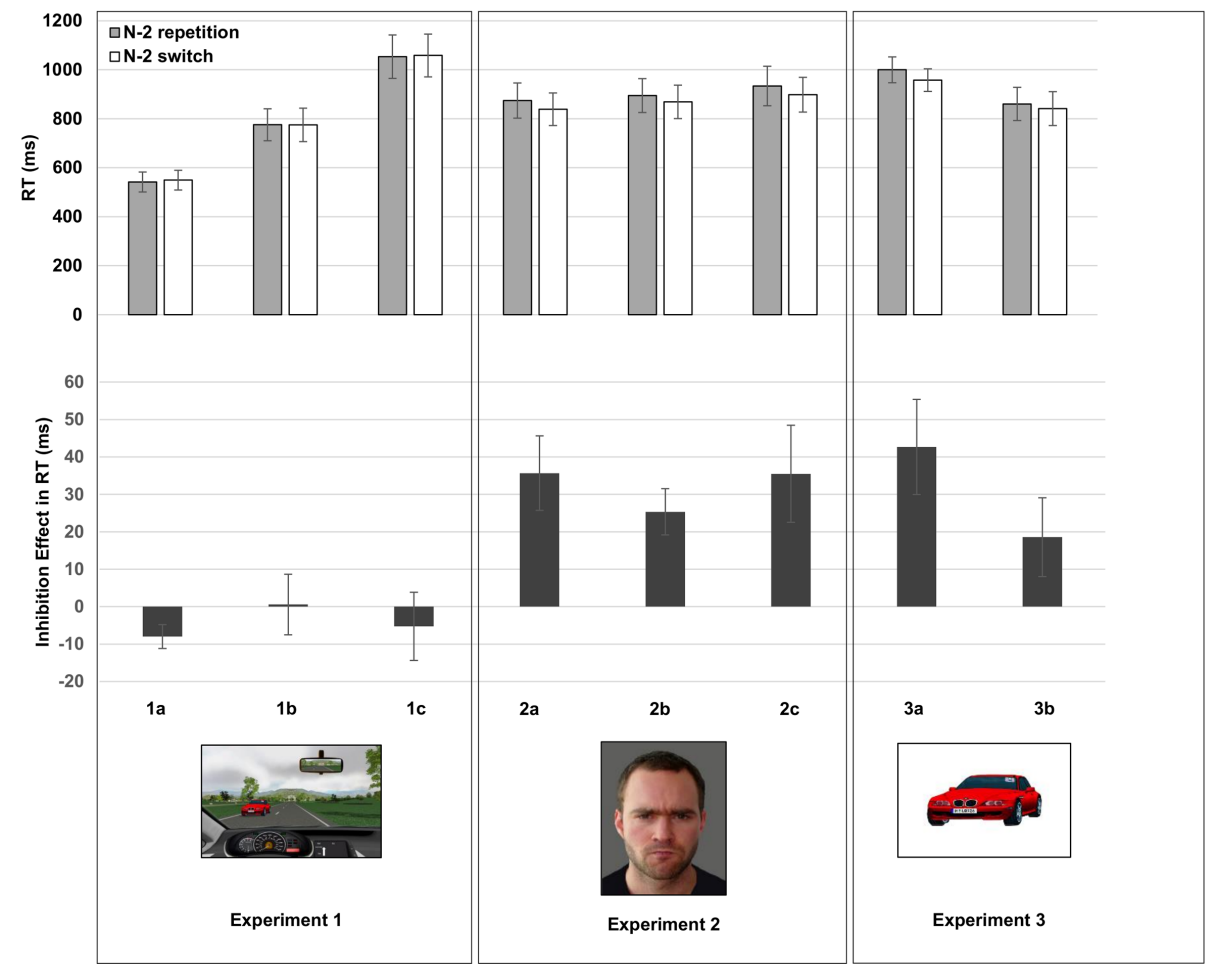
Mean RT in N-2 repetitions and N-2 switches and mean RT inhibition effects in Experiments 1, 2, and 3. Inhibition effects were computed as the difference between N-2 repetitions and N-2 switches. Error bars indicate one standard error of mean. 1a: car tasks, auditory verbal cues; 1b: car tasks, auditory verbal cues; 1c: car tasks, abstract visual cues. 2a: face tasks, cue stays; 2b: face tasks, cue disappears; 2c: face tasks, 8 different stimuli only. 3a: car tasks, abstract visual cues, cars presented centrally on plain background; 3b: car tasks, direct replication.

Mean RT per condition are shown in Figure 2. The ANOVA with the factors Task Sequence and Cue Condition (1a, 1b, 1c) did not yield a significant main effect of Task Sequence, *F*(1,33) = 1.0, and no interaction, *F*(2,33) < 1.0. A significant main effect of Cue Condition, *F*(2,33) = 14.22, *p* < .01, η^2^*_p_* = .46, indicated fastest RTs in Cue Condition 1a (auditory verbal cues, mean RT = 546 ms), intermediate RTs in Cue Condition 1b (visual verbal cues, mean RT = 775 ms), and slowest RTs in Cue Condition 1c (visual abstract cues, mean RT = 1,056 ms). When tested separately, N-2 repetition costs (computed as mean RT in N-2 repetitions minus mean RT in N-2 switches) were –8 ms in Cue Condition 1a, *t*(11) = 2.51, *p* = .03 (i.e., a N-2 repetition *benefit* was obtained in this condition), 1 ms in Cue Condition 1b, *t*(11) = 0.07, *p* = .94, and –5 ms in Cue Condition 1c, *t*(11) = 0.58, *p* = .58.

#### ER

**Figure 3:**
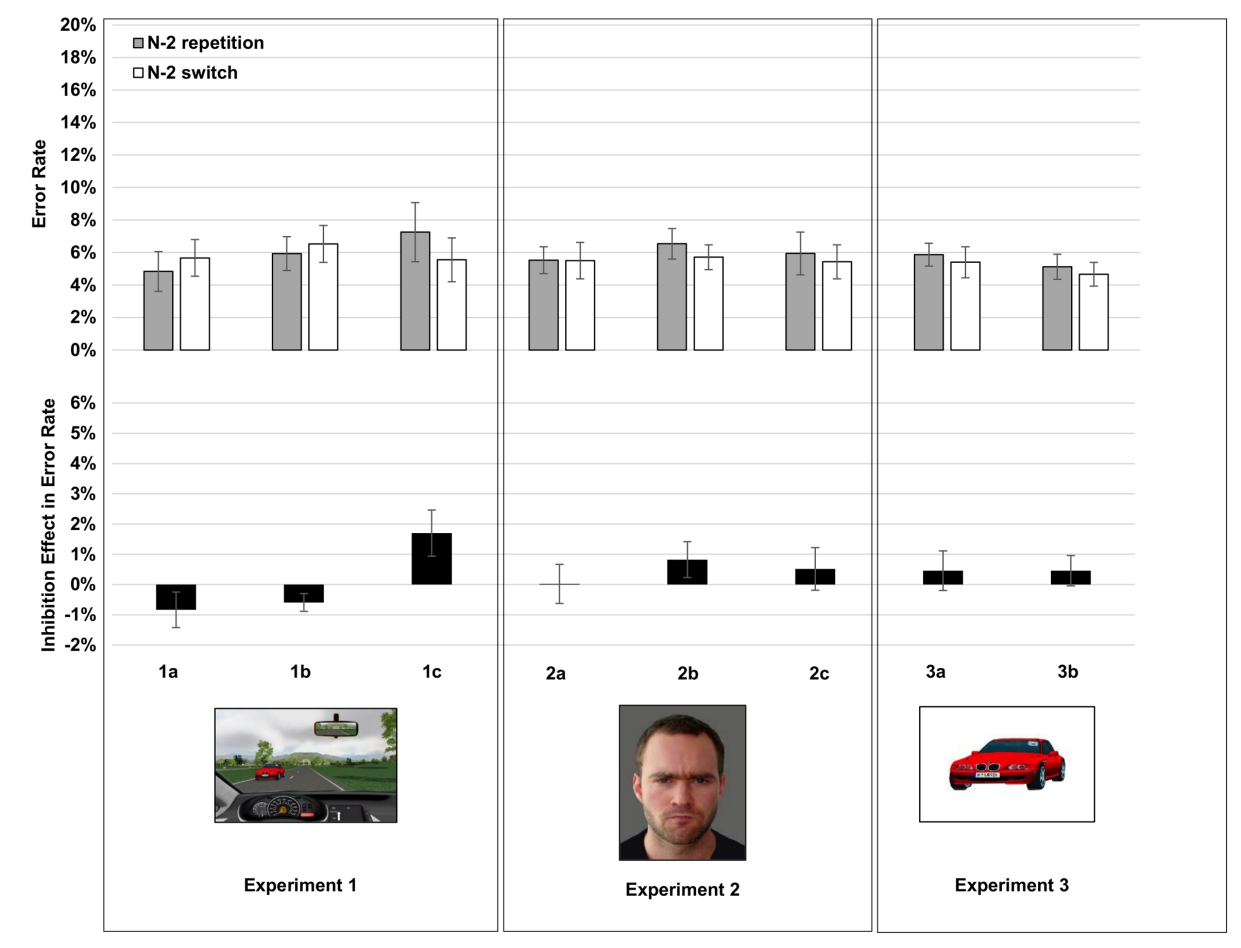
Mean Error Rates in N-2 repetitions and N-2 switches and mean inhibition effects in Experiments 1, 2, and 3. Error bars indicate one standard error of mean. 1a: car tasks, auditory verbal cues; 1b: car tasks, auditory verbal cues; 1c: car tasks, abstract visual cues. 2a: face tasks, cue stays; 2b: face tasks, cue disappears; 2c: face tasks, 8 different stimuli only. 3a: car tasks, abstract visual cues, cars presented centrally on plain background; 3b: car tasks, direct replication.

Mean ER per condition are shown in Figure 3. The ANOVA with the factors Task Sequence and Cue Condition did not yield a main effect of Task Sequence, *F*(1,33) < 1, or Cue Condition, *F*(2,33) < 1. An interaction indicated that N-2 repetition costs differed across the different conditions, *F*(2,33) = 5.76, *p* < .01, η^2^*_p_* = .26. When tested separately, N-2 repetition costs were –0.8% in Cue Condition 1a, *t*(11) = 1.41, *p* = .18; –0.6% in Cue Condition 1b, *t*(11) = 1.99, *p* = .07; and 1.7% in Cue Condition 1c, *t*(11) = 2.23, *p* = .05.

### Discussion

To summarize, we did not observe N-2 repetition costs in RT, and only some very weak indication of N-2 repetition costs in ER. The absence of N-2 repetition costs is surprising, given that the structure of Experiment 1 was very similar to other studies in the cognitive psychology literature, where robust N-2 repetition costs were observed (see Koch et al., 2010, for review): Participants switched between three different categorization tasks on a trial-by-trial basis; the stimuli were multivalent, such that the relevant categorization task cannot be determined on the basis of the presented stimulus, but needs to be inferred from the task cue presented prior to the stimulus; moreover, responses were multivalent, such that the same set of response keys is used for the different tasks. These features are known to produce large interference between the tasks, and therefore provide optimal conditions for observing N-2 repetition costs (Koch et al., 2010; see also Kiesel et al., 2010; Monsell, 2003; Vandierendonck et al., 2010).

One may argue that two aspects of Experiment 1 differ from typical task-switching and N-2 repetition cost paradigms: First, the task cue disappeared upon stimulus onset in Experiment 1, whereas in many task-switching and N-2 repetition cost paradigms, it remains present during stimulus presentation, especially when visual task cues are used. A quickly disappearing task cue might modulate N-2 repetition costs (e.g., Druey & Hübner, 2007), possibly due to an increased working-memory load (cf. Houghton, Pritchard, & Grange, 2010; Mayr & Kliegl, 2000). Second, stimulus-set size was rather small (- only eight different stimuli were used -), making stimulus repetitions relatively frequent. Even though immediate stimulus repetitions from trial N-1 to trial N, and from trial N-2 to trial N, were excluded by design, stimulus repetitions from N-3 to N, N-4 to N, etc. might still have occurred, which might have increased the influence of episodic interference on N-2 repetition costs (see Grange, Kowalczyk, & O’Loughlin, 2017). To address these issues in Experiment 2, we used a paradigm that had produced robust N-2 repetition costs in several previous studies (e.g., Schuch & Grange, 2019; Schuch & Konrad, 2017; Schuch, 2016). We manipulated cue presentation time and number of stimuli, in order to explore whether these features could explain the absence of N-2 repetition costs in Experiment 1.

## Experiment 2

Experiment 2 was structurally identical to Experiment 1, in that participants switched between three different categorization tasks, the relevant task was indicated by a task cue preceding the stimulus, and the stimuli and responses were multivalent. Experiment 2 differed from Experiment 1 with respect to stimulus material and categorization tasks: A face categorization paradigm with abstract visual cues was applied in Experiment 2 that had produced robust N-2 repetition costs in several previous studies (e.g., Schuch & Grange, 2019; Schuch & Konrad, 2017; Schuch, 2016). Across three experimental conditions (between-subjects), we varied cue presentation time and stimulus set size, in order to explore whether these features modulated N-2 repetition costs. In particular, we varied whether or not the task cue remained visible during stimulus presentation (as in our previous studies), or disappeared upon stimulus onset (as in Experiment 1). Moreover, we varied whether the stimulus set consisted of 40 different pictures (as in our previous studies), or 8 different pictures (as in Experiment 1).

Both manipulations might possibly affect N-2 repetition costs: When the task cue disappears upon stimulus onset, participants need to keep the currently relevant task activated in working memory, which might increase working-memory load, and thereby increase N-2 repetition costs (Houghton et al., 2010; Mayr & Kliegl, 2000). Using a smaller set of possible stimuli increases the likelihood that recently presented stimuli are repeated, which in turn could increase the contribution of episodic retrieval effects, and reduce N-2 repetition costs (Grange et al., 2017).

### Method

#### Participants

36 persons participated in Experiment 2 (12 per cue condition 27 females, 9 males, mean age 22.0 years, all were between 18 and 33 years old). None of them had participated in Experiment 1. They received either monetary compensation (6 Euros) or course credits in return for participation.

#### Stimulus material

Standardized photographs of the faces of 40 different adult persons were presented, which differed regarding age (young vs. older adults), gender (male vs. female), and emotional expression (happy vs. angry). Five different persons were presented for each possible combination of young/old, male/female, and happy/angry. All persons were photographed wearing a black t-shirt and no make-up, jewelry, or beards (see Figure 1 for example stimuli). All portraits were edited to a unitary format with identical light grey background (see Schuch, Werheid, & Koch, 2012). The size of the presented faces on the screen was 10.5 cm in width and 14.0 cm in height. Viewing distance was about 40 cm.

#### Task cues

As in our previous studies (Schuch & Grange, 2019; Schuch & Konrad, 2017; Schuch, 2016), each portrait was presented inside a colored frame, with red frame indicating the “age” task, blue frame indicating the “gender” task, and yellow frame indicating the “emotion” task. The inside of the frames measured 10.5 cm in width and 14.0 cm in height (i.e., just the size of the face pictures); the thickness of the frame lines was 0.3 cm.

#### Responses and response mappings

Participants used the same response keys as in Experiment 1. Response mappings were counterbalanced across participants, with half of the participants in each experimental condition responding with the left key to happy, young, and male faces, and with the right key to angry, old, and female faces; the other half of participants received the reverse mapping. A paper card displaying the response mappings was placed below the screen and remained visible throughout the experiment.

#### Experimental procedure

The experimental procedure was identical to that of Experiment 1.

#### Trial procedure

The trial procedure was identical to that of Experiment 1b and 1c in that the CSI was 500 ms, the task cues were presented visually, and there was no time limit for responding. As soon as the correct response key was pressed, the picture disappeared, and after 1000 ms, the next task cue was presented. When the wrong response key was pressed, an error feedback occurred for 1000 ms, and after another 1000 ms, the next task cue was presented.

In Condition 2a, the task cue (colored frame) stayed on the screen while the stimulus (face picture) was presented, and disappeared together with the stimulus as soon as a response key was pressed. This procedure was adopted from previous work with this paradigm (Schuch & Grange, 2019; Schuch & Konrad, 2017; Schuch, 2016).

In Condition 2b, the task cue (colored frame) was only presented during the CSI, and disappeared as soon as the stimulus (face picture) was presented. This procedure was adopted from Experiment 1, where the task cue also disappeared upon stimulus presentation. Everything else was identical to Condition 2a.

Condition 2c was identical to Condition 2b, except that instead of 40 different pictures of faces, only 8 different pictures were used (one for each possible combination of young/old, male/female, and happy/angry face). This was accomplished in order to make Condition 2c as similar as possible to Experiment 1, where we had also used a set of 8 different stimuli.

#### Design

A 2 × 3 mixed design was applied with the within-subject variable Task Sequence (N-2 Repetition, N-2 Switch) and the between-subject variable Condition (Condition 2a: cue remains visible, 40 different stimuli; Condition 2b: cue disappears, 40 different stimuli; Condition 2c: cue disappears, 8 different stimuli).

### Results

#### Data filtering

Data filtering was the same as in Experiment 1. The mean number of trials per participant included in the analysis of Experiment 2 was 278 (min 200, max 321) for RT analysis, and 294 (min 237, max 325) for analysis of Error Rates.

#### RT

The ANOVA with the factors Task Sequence and Condition (2a, 2b, 2c) revealed a robust main effect of Task Sequence, *F*(1,33) = 30.44, *p* < .01, η^2^*_p_* = .48, indicating N-2 repetition costs of 32 ms across all conditions, and no other effects, *F*s < 1. When tested separately, N-2 repetition costs were 36 ms in Condition 2a, *t*(11) = 3.58, *p* < .01; 25 ms in Condition 2b, *t*(11) = 4.09, *p* < .01, and 36 ms in Condition 2c, *t*(11) = 2.74, *p* = .02.

An ANOVA on the combined data from Experiments 1 and 2 with the factors Experiment (1 vs. 2) and Task Sequence yielded a significant interaction, *F*(1,70) = 26.63, *p* < .01, η^2^*_p_* = .28, indicating that N-2 repetition costs were larger in Experiment 2 than Experiment 1. The main effect of Task Sequence was also significant, *F*(1,70) = 15.71, *p* < .01, η^2^*_p_* = .18; the main effect of Experiment was not, *F*(1,70) = 1.99, *p* = .16.

#### ER

The ANOVA with the factors Task Sequence and Condition did not yield any significant effects (*F*(1,33) = 1.48 for the main effect of Task Sequence; all other *F*s < 1). When tested separately, N-2 repetition costs were 0.0% in Condition 2a, *t*(11) = 0.04, *p* = .97; 0.8% in Condition 2b, *t*(11) = 1.4, *p* = .19; 0.5% in Condition 2c, *t*(11) = 0.73, p = .48. An ANOVA on the combined data from Experiments 1 and 2 did not yield any significant effects, *F*s(1,70) < 1.1.

### Discussion

To summarize, in Experiment 2, we observed robust N-2 repetition costs in RT, in line with earlier studies in the literature. N-2 repetition costs in Experiment 2 were significantly larger than those in Experiment 1. We did not observe any indication that N-2 repetition costs were modulated by cue presentation time or stimulus set size in Experiment 2. We note, however, that this experiment was not designed to detect small modulations of N-2 repetition costs, because a between-subject design with relatively small group sizes was used. Importantly for the present context, we observed significant N-2 repetition costs in each of the conditions 2a, 2b, and 2c. This indicates that cue presentation time or stimulus set size are unlikely to have caused the lack of N-2 repetition costs in Experiment 1.

Another feature of Experiment 1 that might have caused the absence of N-2 repetition costs was that the stimuli were embedded in a visual scene that contained distractors: Apart from the task-relevant oncoming car, the pictures also contained the road ahead, the surrounding landscape, as well as parts of the dashboard and back mirror as seen from the driver’s perspective (see Figure 1). Moreover, the task-relevant oncoming car was not presented centrally, but lateralized. In order to explore whether distractor presence and/or lateralized presentation caused the lack of N-2 repetition costs in Experiment 1, we conducted another experiment where we used the same task-relevant stimuli (i.e., the pictures of the oncoming cars), but presented them without any distractors, and placed centrally on the screen (see Figure 1).

## Experiment 3

### Method

#### Participants

24 new persons participated in Experiment 3 (12 each in Conditions 3a and 3b). 19 were female, 5 males, mean age 22.6; all were between 18 and 29 years of age). They were either students, or friends of students, of RWTH Aachen University, and received either monetary compensation, or course credits, or sweets in return for participation.

#### Stimulus material

The stimuli were the eight oncoming cars from the stimulus material of Experiment 1, but this time, they were presented centrally on the screen on white background (see Figure 1). The pictures were of width 14.0 cm and height 10.5 cm (i.e., the size of the pictures was identical to that in Experiment 2, except that they were in landscape format instead of portrait format; see Figure 1). Viewing distance was about 40 cm.

#### Task cues

The task cues were white frames, with the inside of the frame measuring 14.0 cm in width and 10.5 cm in height (i.e., just the size of the car pictures). The frames differed with respect to the thickness of the horizontal and vertical lines, which could be of either 0.3 cm or 1.0 cm thickness. As in Condition 1c, the color task was indicated by a frame with thin horizontal and thin vertical lines, the brand task by a frame with thick horizontal lines, the toll-sticker task by a frame with thick vertical lines.

#### Responses

The response set, response mappings, and counterbalancing of response mappings were identical to Experiment 1.

#### Experimental procedure and Trial procedure

These were identical to those of Conditions 1b, 1c and Experiment 2.

#### Design

A 2 × 2 mixed design was applied with the within-subject variable Task Sequence (N-2 Repetition, N-2 Switch) and the between-subject variable Condition (Group 1, Group 2). Group 2 was a direct replication of Group 1 (- in order to make the design as similar as possible to Experiments 1 and 2, we still included Condition as a variable). As with Experiment 2, we were mainly interested in the main effect of Task Sequence, that is, whether N-2 repetition costs can be observed with these kinds of stimuli, and whether N-2 repetition costs differ significantly between Experiment 3 and Experiment 1.

## Results

### Data filtering

Data filtering was the same as in Experiments 1 and 2. The mean number of trials per participant and condition included in the analysis of Experiment 3 was 280 (min 216, max 320) for RT analysis, and 296 (min 244, max 326) for analysis of Error Rates.

### RT

The ANOVA with the factors Task Sequence and Condition (3a, 3b) yielded a main effect of Task Sequence, *F*(1,22) = 13.75, *p* < .01, η^2^*_p_* = .39, indicating robust N-2 repetition costs of 31 ms across Conditions 3a and 3b, and no other effects, *F*s (1,22) < 2.3. When tested separately, N-2 repetition costs were 43 ms in Condition 3a, *t*(11) =3.35, *p* < .01, and 19 ms in Condition 3b, *t*(11) = 1.76, *p* = .11.

An ANOVA on the combined data from Experiments 1 and 3 with the factors Experiment (1 vs. 3) and Task Sequence yielded a significant interaction, *F*(1,58) = 16.65, *p* < .01, η^2^*_p_* = .22, indicating that N-2 repetition costs were larger in Experiment 3 than Experiment 1. The main effect of Task Sequence was also significant, *F*(1,58) = 9.56, *p* < .01, η^2^*_p_* = .14; the main effect of Experiment was not, *F*(1,58) = 2.84, *p* = .10, η^2^*_p_* = .05.

### ER

The ANOVA on Error Rates did not yield any significant effects, all *F*s(1,22) < 1.25. N-2 repetition costs were 0.5%, *t*(11) = 0.70, *p* = .50, in Condition 3a, and 0.5%, *t*(11) = 0.91, *p* = .38, in Condition 3b. An ANOVA on the combined data from Experiments 1 and 3 with the factors Experiment (1 vs. 3) and Task Sequence did not yield any significant effects, either, all *F*s < 1.

### Analysis of variability in Experiments 1, 2, and 3

Since Experiment 1 employed more complex scenes than Experiment 2 and 3, it is conceivable that the lack of N-2 repetition costs in Experiment 1 was due to higher data variability. To find out, we analyzed the intra-individual and inter-individual variability in all experiments and conditions (see Table 1). Intra-individual variability of RT data was calculated as the standard deviation of all valid raw RT trials per participant and condition (N-2 repetition, N-2 switch), and then averaged across participants in each experimental group. Inter-individual variability of RT was calculated as the standard deviation of participants’ mean RT in each experimental group and condition (N-2 repetition, N-2 switch). Inter-individual variability of error data was calculated accordingly.

**Table 1:** Analysis of variability in Experiments 1, 2, and 3. Intra-individual variability of RT data, and inter-individual variability of RT and error data. Scores are standard deviations of all participants in each experimental group and condition. 1a: car tasks, auditory verbal cues; 1b: car tasks, auditory verbal cues; 1c: car tasks, abstract visual cues. 2a: face tasks, cue stays; 2b: face tasks, cue disappears; 2c: face tasks, 8 different stimuli only. 3a: car tasks, abstract visual cues, cars presented centrally on plain background; 3b: car tasks, direct replication.

	Experiment 1			Experiment 2			Experiment 3	

	1a	1b	1c	2a	2b	2c	3a	3b

Intra-individual variability in RT (in ms)								
N-2 repetition	158	342	502	413	377	393	544	446
N-2 switch	162	349	514	390	357	380	524	441
Inter-individual variability in RT (in ms)								
N-2 repetition	135	216	294	238	230	268	175	226
N-2 switch	134	226	288	221	225	235	153	230
Inter-individual variability in Error Rates								
N-2 repetition	4.1%	3.4%	6.0%	2.7%	3.1%	4.4%	2.3%	2.6%
N-2 switch	3.7%	3.8%	4.5%	3.7%	2.5%	3.5%	3.2%	2.4%

The outcome, summarized in Table 1, provides no indication of larger variability in Experiment 1 relative to Experiments 2 and 3. We thus have no evidence that the lack of N-2 repetition costs in Experiment 1 was due to higher data variability.

## General Discussion

We report a series of three experiments exploring inhibition in task switching, using N-2 repetition costs as an empirical marker. In all experiments, participants switched between three different categorization tasks, corresponding to standard paradigms used in cognitive psychology. The experiments were structurally very similar, but differed with respect to the stimulus material.

In Experiment 1, we used static pictures of driving scenes that were taken from a virtual reality driving setting. The pictures showed oncoming cars on a road embedded in a landscape, from the perspective of a driver’s seat. Participants categorized the oncoming cars according to three different features of these tasks (color, brand, sticker on windshield). To our surprise, we did not observe N-2 repetition costs, no matter whether the tasks were cued auditorily or visually, and whether they were cued by semantic or abstract cues (except for an effect in error rates, but not in RT, in the condition with abstract cues). In Experiment 2, we used static pictures of centrally presented faces on light-grey background, and observed robust N-2 repetition costs in RT data, no matter whether the task cue stayed on the screen or disappeared upon presentation of the face, and whether a large or small set of different faces was used. In Experiment 3, we used the pictures of cars from Experiment 1, but without the surrounding visual scene, and presented centrally on plain background. In contrast to Experiment 1, we observed robust N-2 repetition costs in the RT data of Experiment 3.

On the basis of these results, we conclude that N-2 repetition costs depend on the complexity of the stimulus material. N-2 repetition costs were obtained when the task-relevant stimuli were displayed without a surrounding visual scene, but not when the task-relevant stimuli were part of a complex visual scene. We conjecture that the visual scene might have activated additional task sets, and/or that the presence of distractor stimuli and the lateralized presentation of the relevant stimulus within the scene might have triggered shifts of spatial attention. Both additional task sets and shifts of spatial attention might have attenuated N-2 repetition costs.

As a caveat, it is notoriously difficult to conclude that an effect does not exist under certain conditions. As a reviewer pointed out, it is possible that the more complex visual stimuli used in Experiment 1 lead to increased noise in the data, which might have contributed to not finding significant N-2 repetition costs in Experiment 1, even though the cognitive inhibitory mechanism might still have been in place. In order to explore this possibility, we additionally analyzed the variability of the data in all experiments. We did not observe any evidence for increased variability in Experiment 1 relative to Experiments 2 and 3, suggesting that the noise in the data was comparable across the experiments. In the following, we will further elaborate the possibility that the more complex visual scenes used in Experiment 1 might have triggered additional task-sets and additional shifts of spatial attention.

### Stimulus complexity might trigger additional task-sets

In Experiment 1, the relevant stimulus (i.e., the oncoming car) was embedded in a complex visual scene, and this might have led to additional cognitive processing of distractor stimuli. The view from the driver’s seat of a car, as presented in the stimuli of Experiment 1, is probably a familiar situation to most participants. Several aspects of the scene might attract attention in skilled drivers, for instance, the back mirror and the control displays on the dashboard, such as speed indicator and fuel indicator. These distractor stimuli might trigger additional task sets that were not instructed. For instance, participants might have checked the traffic situation via the back mirror, or checked the dashboard for unexpected warning stimuli lighting up. Thus, participants might have performed more tasks than just the instructed ones, and therefore more task switches might have occurred during a sequence of three instructed tasks, leading to an attenuation of N-2 repetition costs.

### Stimulus complexity might trigger shifts of spatial attention

The task-irrelevant aspects of the visual scene might have attracted attention either covertly (i.e., without eye movements) or overtly (i.e., by triggering eye movements towards them). Covert shifts of spatial attention triggered by non-focal stimulus features (i.e., features that are not at the center of fixation) have been vastly investigated in cognitive psychology with the help of Posner’s spatial cuing paradigm and its derivatives (Posner, 1980; for reviews, see Carrasco, 2011; Müller & Geyer, 2009; Spence & Santangelo, 2009). Overt shifts of attention – that is, eye movements – might have been triggered not only by non-focal distractors, but also by the non-focal spatial position of the relevant stimulus feature (oncoming car). In all pictures of Experiment 1, the oncoming car was not presented at the center of the scene, but lateralized to the left. Moreover, the overall size of the pictures in Experiments 1a and 1b was larger than that in Experiments 2 and 3, which might also have triggered eye movements.

At this point, we can only speculate in what way covert and overt shifts of spatial attention might have interfered with the cognitive control process of task-set inhibition. A recent study by Hilt and Cardellicchio (2020) reported an interaction between shifts of spatial attention and motor inhibition, as measured by the stop-signal paradigm. They found that a shift of spatial attention increased the latency of stopping, suggesting that an attention shift impaired motor inhibition. Possibly, similar interactions occur between attention shifts and cognitive inhibition, as measured with N-2 repetition costs. Moreover, neuroscientific evidence suggests that stimulus-triggered shifts of attention interrupt ongoing task-related processing, and lead to goal-directed re-orientation of attention (Corbetta & Shulman, 2002).

A modulation of N-2 repetition costs by spatial attention processes was also observed by Arbuthnott and colleagues (Arbuthnott & Woodward, 2002; see also Arbuthnott 2005, 2008, 2009). These authors reported diminished N-2 repetition costs when the different tasks were presented in separate spatial locations. In their experimental paradigm, the spatial position of a visually presented task cue indicated the relevant task, and the to-be-categorized stimulus was subsequently presented at the same location. Arbuthnott and colleagues suggested that the spatial separation of the tasks reduced interference between tasks, leading in turn to smaller N-2 repetition costs. Other than in Arbuthnott’s work, in the present experiments, the task cue and stimulus were always presented in the same spatial location. Hence, reduced interference due to spatial separation of the tasks cannot explain the present results. Rather, we suggest that the more complex stimulus material caused additional interference, by triggering additional shifts of spatial attention, and additional distractor processing.

### Laboratory settings versus real-life situations

The present results provide an example of the difficulties that may arise when aiming to transfer laboratory paradigms to more applied contexts. Already small changes in the stimulus layout (- in the present experiments, the presence of distractors and/or the non-central presentation of the relevant stimulus feature -) can eliminate effects that are reliably obtained when simple visual stimuli are used in typical laboratory paradigms.

We note that the step towards a more applied setting was rather small in the present study. In contrast to natural car driving, we used static screenshots rather than dynamic visual scenery, disregarded the effects of drivers’ own actions on their perception (see, e.g., Engel, Friston, & Kragic, 2016; Warren, 2006, for action-oriented views of cognition), and also disregarded the cognitive load of movement planning (e.g., Beurskens & Bock, 2012; Krampe, Schaefer, Lindenberger, & Baltes, 2011; Woollacott & Shumway-Cook, 2002), as required to control the car’s speed and lane position. We also disregarded that natural driving is a highly complex multitasking situation: besides playing car-naming games and controlling the car’s movement, drivers must observe traffic signs, watch for other cars, pedestrians and obstacles on the road, plan and keep track of the route, etc. It is well established that multitasking can degrade performance on the component tasks (e.g., Koch, Poljac, Müller and Kiesel, 2018), and even is a major contributing factor for car accidents (e.g., Dingus, Guo, Lee, Antin, Perez, Buchanan-King and Hankey, 2016). Finally, we disregarded that natural driving typically involves desirable goals: drivers play car-naming games to enjoy the company of their passengers, rather than to comply with an experimenter’s instructions. To conclude, more work needs to be done to determine how factors like dynamic perception, cognitive load, multitasking and goal-setting affect robust laboratory findings, such as n-2 repetition costs.

## Data Accessibility Statement

Raw data are available on the Open Science Framework: https://osf.io/jez7h/.
